# Effect of Material and Process Specific Factors on the Strength of Printed Parts in Fused Filament Fabrication: A Review of Recent Developments

**DOI:** 10.3390/ma12101664

**Published:** 2019-05-22

**Authors:** Muhammad Harris, Johan Potgieter, Richard Archer, Khalid Mahmood Arif

**Affiliations:** 1 School of Food and Advanced Technology, Massey University, Auckland 0632, New Zealand; m.harris@massey.ac.nz; 2 Massey Agritech Partnership Research Centre, Massey University, Palmerston North 4442, New Zealand; j.potgieter@massey.ac.nz; 3 School of Food and Advanced Technology, Massey University, Palmerston North 4442, New Zealand; r.h.archer@massey.ac.nz

**Keywords:** fused deposition modeling, materials, parameters, voids, elasticity, tensile strength

## Abstract

Additive manufacturing (AM) is rapidly evolving as the most comprehensive tool to manufacture products ranging from prototypes to various end-user applications. Fused filament fabrication (FFF) is the most widely used AM technique due to its ability to manufacture complex and relatively high strength parts from many low-cost materials. Generally, the high strength of the printed parts in FFF is attributed to the research in materials and respective process factors (process variables, physical setup, and ambient temperature). However, these factors have not been rigorously reviewed for analyzing their effects on the strength and ductility of different classes of materials. This review systematically elaborates the relationship between materials and the corresponding process factors. The main focus is on the strength and ductility. A hierarchical approach is used to analyze the materials, process parameters, and void control before identifying existing research gaps and future research directions.

## 1. Introduction 

Additive manufacturing (AM) or 3D printing [1,2,3,4,5] is the next generation manufacturing technology that allows manufacturing of complex parts without requiring specialized tooling [6,7]. Therefore, AM is currently being used in a wide range of applications such as high value consumer products [8], food [9], electronics [10,11,12], machinery [13], aerospace industry [14,15], automobiles [16,17], medical and dental applications [4,18,19,20,21,22], textile [23,24,25], construction [26,27,28,29], education [30], and architecture [31,32,33,34,35,36,37,38,39,40]. AM has various forms that share the same concept of layer-by-layer manufacturing [30,40,41,42,43,44]. The most common among all the AM technologies is fused filament fabrication (FFF), also known as fused deposition modeling (FDM). In FFF, melt thermoplastic polymers are extruded to make the layers for fabricating the design provided in one of the following formats: STL (stereolithography), AMF (additive manufacturing file), Step (standard for exchange of product model data), Voxel, 3MF (3D manufacturing format), or JT (Jupiter tessellation) [45,46,47,48]. The simple extrusion process that can be applied to a large variety of materials makes FFF an affordable technology for research institutes, industries and domestic users. 

Despite the technical simplicity, geometric accuracy, ability to build complex shapes with no waste of material and commercial success [49], the FFF structure is composed of voids that contribute to the vulnerability of the product to lack mechanical properties [50]. In the recent growing market, structural integrity is represented by numerous characteristics like strength, fatigue resistance, resistance to aging, resistance to chemical and moisture erosion, etc. Major research in FFF encompasses numerous facets that aim to improve strength (tensile, compressive, and flexural), ductility, and modulus (elastic and flexural) [50,51,52,53,54,55,56]. Since the invention of FFF/FDM in the 20th century, researchers have adopted different ways to improve the strength of parts. However, for a long time, FFF research remained limited to the process parameters/variables (feed rate, speed, layer thickness, etc.) and single materials [57]. In this regard, a recent review by Cuan-Urquizo et al. [58] presents a comprehensive overview of the effect of process parameters on the mechanical properties of FFF parts. 

**Figure 1:**
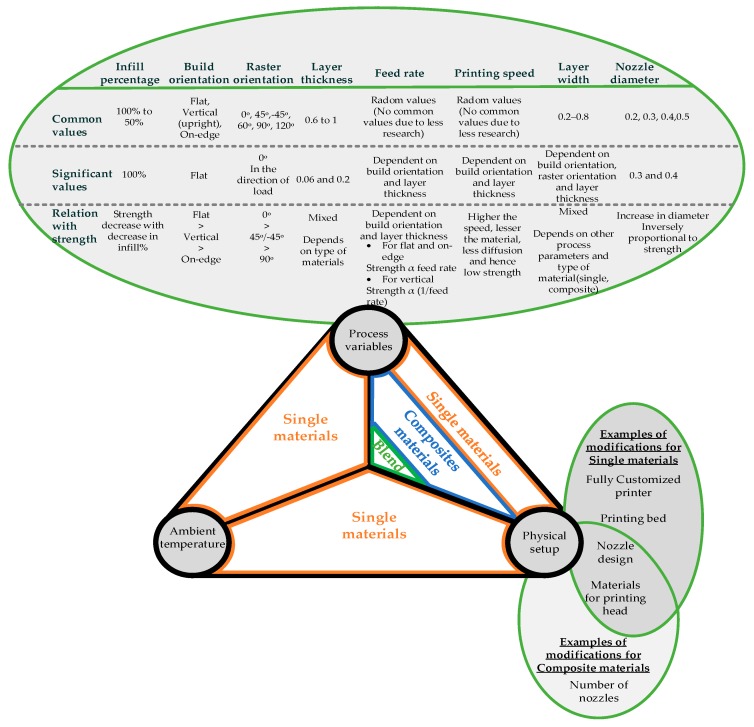
Summary of fused filament fabrication (FFF) materials and corresponding process factors (process variables, physical setup modifications, and controlled ambient temperature) based on the overall understanding of literature [1,57,58,59,60,61,62,63,64,65,66,67,68,69,70,71,72].

It has been observed that the advancements in the form of customized physical setups, ambient temperature control, and different forms of new materials (composites and blends) have become the main research topics to enhance strength in the last decade or so. Therefore, the strength of printed parts is not only a result of process parameters but is also affected by the material construct (not just the material strength), in addition to process specific strategies and factors. In Figure 1, the vertices of the equilateral triangle identify the three most important factors: (1) process variables, (2) physical setup, and (3) ambient temperature control. The triangle is also divided into three equal sections, each one showing material construct that is related to the vertices on the line touching the particular section. The factors on the vertices for the section of the triangle and the circle together impact the strength of the printed parts. For example, for a single material, process variables and ambient temperature are governing factors to achieve high strength. Similarly, for blends the governing factors are process variables and physical setup.

It is important to mention that there are other types of in-process and post-printing processes performed on the FFF materials. For example, humidity exposure (water absorption) [73,74], chemical treatment (acetone, ethyl acetate, tetrahydrofuran, dichloromethane, chloroform) [75,76,77,78], plasma treatment and epoxy infiltration [79], aluminium coating, metal coating [80,81], physical vapor deposition (PVD) [82], etc. However, apart from humidity [74], these processing techniques are mostly applied for decreasing the surface roughness or to achieve higher dimensional accuracy. Moreover, the before-mentioned post processing techniques are not intended for enhanced tensile strength and ductility. For example, the humidity decreases the strength instead of increasing it. On the contrary, thermal processing (pre-printing, in-process, and post-printing) can result in significant enhancement in tensile strength and ductility [83,84]. 

**Figure 2:**
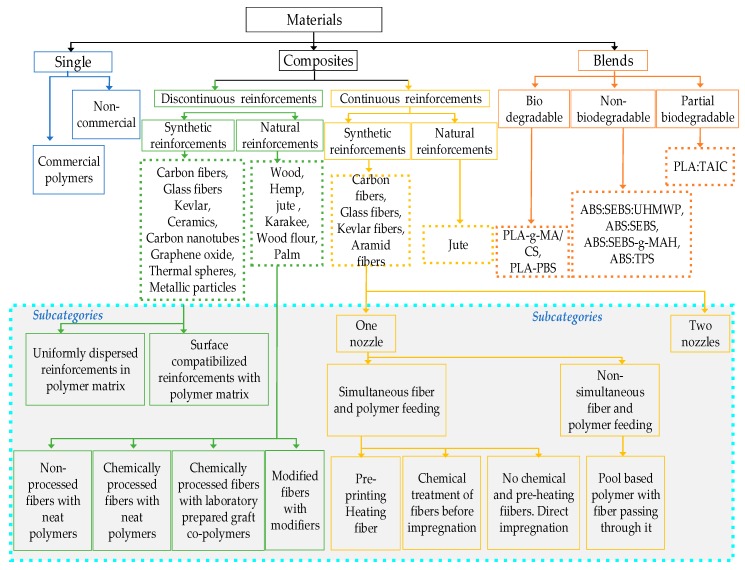
Materials for fused filament fabrication.

This review is focused on the combined effect of materials and process factors on the optimal strength of printed parts. The materials are categorized into three groups: single materials, reinforced composites, and blends. A comprehensive review of each category is provided by dividing the main material category into subcategories in a hierarchical manner (see Figure 2). The approach taken for grouping (subcategorization) is based on the way they are presented in the literature, e.g., commercial and non-commercial materials, partial or fully biodegradable, or continuous and discontinuous materials, etc. Since all the material groups differ from each other, the subcategorization is not the same for each material. For example, single materials are subcategorized into commercial and non-commercial categories. On the other hand, blends are presented as multi-layered subcategories to capture the correct terminology and relevance in the context of FFF.

## 2. FFF Materials 

Various materials are researched in FFF, however, not all materials are researched for tensile strength and ductility (Table 1). 

For example, the materials shown in Table 1 have not been specifically investigated for the tensile strength or ductility, e.g., the porous ceramic materials are mostly investigated in terms of compression strength [95,103]. The focus of this review is limited to the materials that are analyzed in terms of tensile strength and elastic modulus. FFF materials are found in three main categories (Figure 2), i.e., single materials, reinforced composites, and blends. 

### 2.1. Single Materials

**Figure 3:**
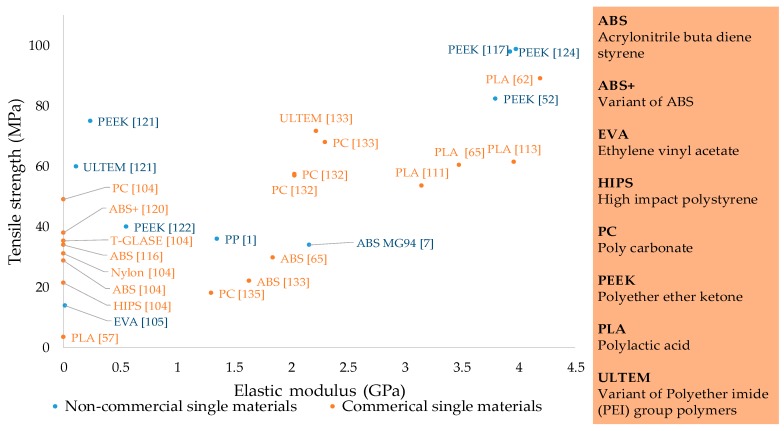
Tensile strength and elastic modulus of single materials of fused deposition modeling.

Single materials are highly significant for FFF as they are commonly available for domestic users. Specifically, the entry level printers (Tiertime [83], Makerbot, Reprap [65], etc.) come with single material filament spools. The single materials presented in the literature include either commercial 3D printing filaments [104] or research-based filaments prepared from polymers by extrusion or injection molding [105]. However, it is noticed that the commercial filaments are used in the majority of research as shown in Figure 3. The reason for this can be the excessive processing requirements and chemistry involved in the filament-making process. Another reason for less research on laboratory-prepared filaments (non-commercial) can be the nature of research that aims for quality of parts only through parametric optimization instead of improvements in the printing materials.

**Figure 4:**
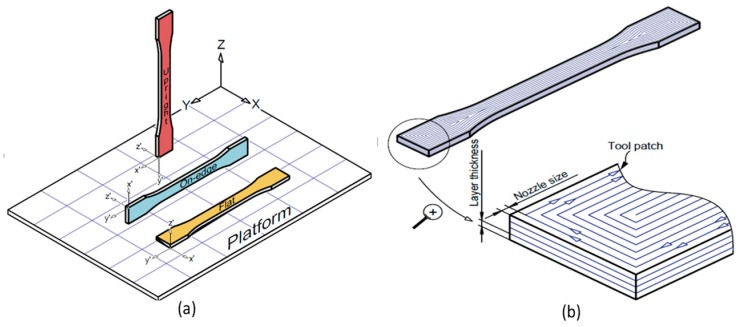
Process parameters: (**a**) build orientation; and (**b**) raster orientation. Adapted from [62], with permission from © 2017 Elsevier.

Various single materials have been researched since the invention of FFF as shown in Table 2 and Figure 3. Significant enhancements have been made in mechanical properties like tensile strength, ductility, and elastic modulus of these materials, specifically in the last couple of years. The enhancements in properties of single materials have been achieved through advancements made in physical setups, optimal process parameters, or controlled environmental conditions. Figure 3 includes the materials that show prominent tensile strength. However, there are other FFF single materials in the literature like high impact polystyrene (HIPS), polycaprolactone (PCL) [92,106], polyvinyl alcohol (PVA) [107], and Polyurethane (TPU) [92], that have been mostly reported for medical applications and exhibit low tensile strength [92]. This section highlights the tensile capability of various potential FFF single materials along with the special measures (e.g., Orientation in Figure 4) taken to achieve enhanced mechanical properties. 

Polylactic acid (PLA) is a renewable, low cost, low melting temperature, and commonly available FFF polymer. It is a biodegradable polymer made by lactic acid or dimers of D-lactides or L-lactides produced by fermentation of starch obtained from natural sources such as plants. It inherits a problem of low crystallization even with the optimal contents of D-lactide (0.5% to 12%) that deprives PLA of achieving good mechanical properties. Various techniques including the addition of additives and post-process thermal treatments have been employed to improve the mechanical properties. The tensile strength gained from PLA structures ranges from 15.5 MPa to 89.1 MPa [41,64,72,108,109,110,111,112]. The highest value of 89.1 MPa, reported in the literature, was achieved with a commercial filament on a small open source 3D printer through an optimal combination of feed rate, layer thickness, and build orientation (Figure 3 and Table 2). This research employed an optimal method of load application in the direction of tool path as shown in Figure 4. The reported reasons for fracture are the inter-layer fusion and trans-layer failure that brought significant difference in strength with the change in build orientation. Inter layer fusion is the fusion bond of lower layer with extruded one and the trans-layer fusion bond is the fusion between beads (roads) of the same layer. In upright (vertical) samples, the breakage of the inter-layer fusion bond occurred due to the applied load parallel to the deposited layer. The deposited layer withstood the whole force instead of individual beads leading to low strength and hence the failure occurred between layers (interlayer). On the contrary, the load applied perpendicular to the deposited layer, in flat and on-edge samples, making the beads bear the applied load, resulted in high strength [62]. 

**Figure 5:**
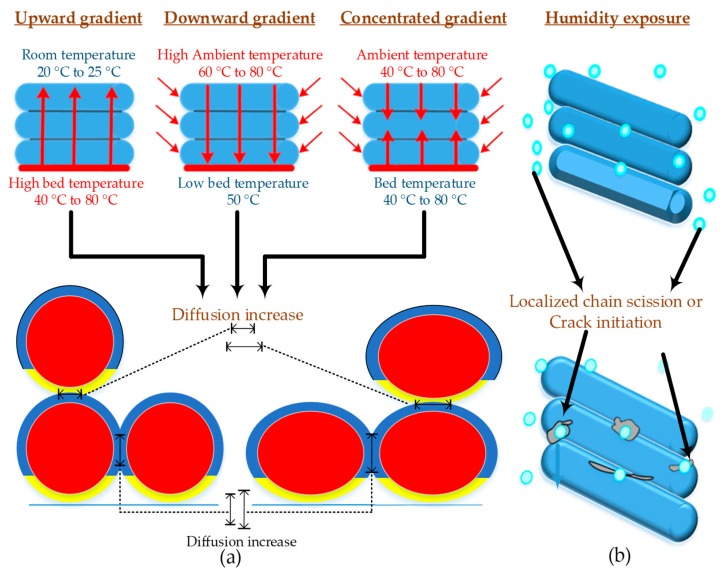
Illustration of in-process and post-process processing on printed structure of PLA: (**a**) in-process thermal treatment at different types of heat gradients; and (**b**) effect of moisture on localized areas.

Another research reports optimal values of strain rate (2.5 × 10^−4^ s^−1^) and raster angle (45°) to achieve 61.4 MPa of tensile strength [113]. PLA is also reported with in-process [83] and post-printing (annealing) thermal treatments. However, it is noted that the annealing does not result in any improvement in tensile properties [113,114]. The in-process thermal treatment reports enhancement in either tensile strength or ductility based on the direction of the heat gradient [83]. However, as shown in Figure 5a, the chemical degradation of PLA has not been reported. Another important aspect researched for PLA is the resistance to humidity. There is a limited literature on behavior of FFF-printed PLA against humidity that reports chain scission (or material separation) at localized areas exposed to moisture (Figure 5b). The increase in percentage humidity causes the tensile load to decrease significantly [73]. The brief aforementioned literature concludes that the optimal process parameters are more in trend to gain high strength for PLA as compared to the controlled environmental conditions or precise physical setups as shown in Table 2. However, the significant degradation of mechanical properties point towards the importance of considering humidity in future PLA 3D printing. 

Acrylonitrile butadiene styrene (ABS) is the only ternary polymer in FFF. It is the most common elastomeric semi-crystalline polymer used for FFF that is considered one of the large-scale materials due to the introduction of entry level printers for domestic users. The tensile strength obtained from commercial ABS filaments ranges from 26 MPa to 38 MPa [7,42,115,116,117,118,119]. The highest strength (38 MPa) is reported for a commercial variant of ABS (ABSplus) for flat build orientation [120]. Multi-purpose injection molding grades of ABS (MG47 and MG94) are also used to extrude out filaments for FFF. A tensile strength of 34 MPa for high molecular weight MG47 at high feed rate (60 mm/s) has been achieved [7]. 

**Figure 6:**
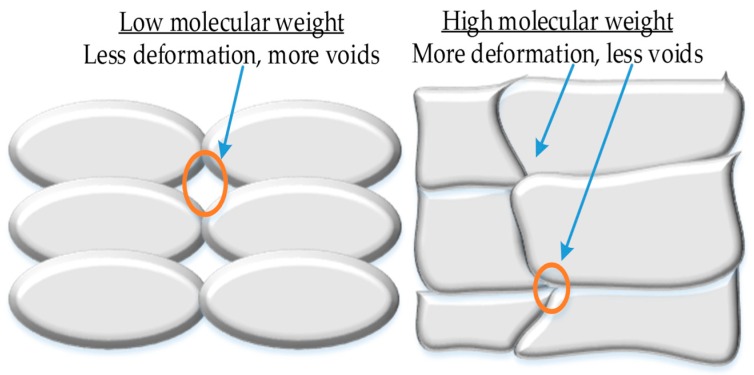
Illustration of effects of molecular weight on diffusion.

The effect of molecular weight is observable in SEM structure as illustrated in Figure 6 that shows high deformation for high molecular weight grade resulting in more fusion area among beads compared to less deformation for low molecular weight grade that causes less fusion area. Therefore, the research reveals the effectiveness of molecular weight for ABS [7]. Moreover, there is a recent research on acetone treatment of ABS in between layers during 3D printing that reports minor improvement in properties [44]. The above-mentioned literature also elaborates on the ability of ABS to generate desired results at an uncontrolled environment as presented in Table 2. 

Polyetherether ketone (PEEK) has recently been reported as a potential research material [56,117,121]. However, it is the most difficult material to 3D print among all FFF materials [122]. The main reason for this is the highest melt temperature (>350 °C) among all FFF polymers that makes its processability extremely difficult. Another problem is the narrow optimal temperature range for successful printing (360–400 °C) [56,121,123]. FFF printing of PEEK has been performed on three kinds of custom-made physical setups: (1) syringe-based [121], (2) extrusion-based [124], and (3) filament-based [125]. Among these setups, screw-based extrusion achieves the highest tensile strength (≈100 MPa) [117], while the filament-based setups show the least strength (40 MPa) [122]. The syringe-based setup is conducive to only low molecular weights and it is reported to achieve incomplete printing [121]. Unlike other FFF materials, research on PEEK utilizes a common grade (Victrex 450G) with high molecular weight [52,117,124] that provides superior strength and elastic modulus with 45°/−45° raster orientation [117]. 

**Figure 7:**
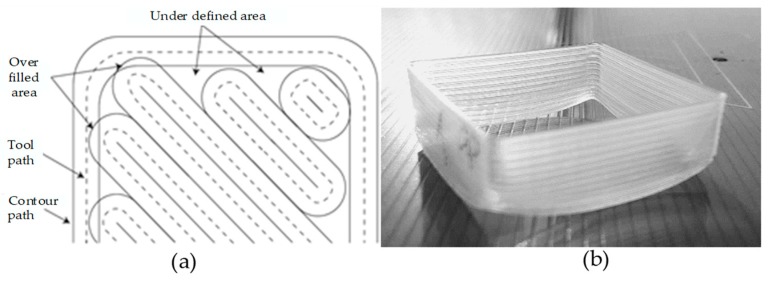
Illustration of PP 3D printing: (**a**) over-filled infill with contour overlap, and (**b**) shrinkage in PP printed samples. Adapted from [1], with permission from © 2015 Elsevier.

Polypropylene (PP) is the only polyolefin in FFF materials in a short list of known FFF materials. It suffers excessive warpage and shrinkage (Figure 7) that produce dimensional instabilities making its printing a challenging job [1,54,127,128,129]. To overcome the printability problems associated with warpage and shrinkage as shown in Figure 7b, different techniques have been employed. For example, retrofitting PP sheet on a non-heated bed, managing the overlapping area between beads through calculating the shrinkage of each layer [54], addition of fibers [1,54] and alcohol treated PP plate scrubbed with steel brush [1] are among the recently reported techniques. The commercial filament of PP is not available and, therefore, researchers use conventional polymer grades like extrusion molding grades. One of the rare works on PP used high feed rate, low printing (plotting) speed along with over-filled infill rasters on contour paths (Figure 7a) extruded through a custom designed extrusion head. The setup helped to achieve 36 MPa at 0° raster angle with constant layer thickness and 100% infill [1]. The literature doesn’t provide any information regarding 3D printing of PP in controlled environments (thermal or vacuum) or in high precision commercial printers, which leaves a research gap to further explore. 

Polycarbonate (PC) is another FFF thermoplastic that has been reported to possess better mechanical properties compared to ABS. The major part of the research performed on PC is on the analysis of tensile properties [130], flexural properties [131], creep [132], and roughness [133] through modifications in process parameters like build orientation, layer thickness, raster angle, number of contours, air gap, etc. [130,131,132,133,134]. The literature predominantly utilizes similar commercial FFF setups (Fortus MC400 and MC360) that fabricate in a heated environment [130,131,132,133], except for one reference that reports both pre-conditioning (ASTM D618) and in-process thermal treatment of PC (vender not provided) [135]. The tensile strength in the literature for PC ranges from as low as 18 MPa [135] to as high as 68 MPa [133]. The recent inclination of research combines the aforementioned process parameters with cyclic or fatigue analysis in heated and non-heated environments [131,132]. It is worth exploring to analyze the effects on mechanical properties of neat (virgin) PC as it is still to be explored. The research will help to explore the real potential of neat polymer. 

**Figure 8:**
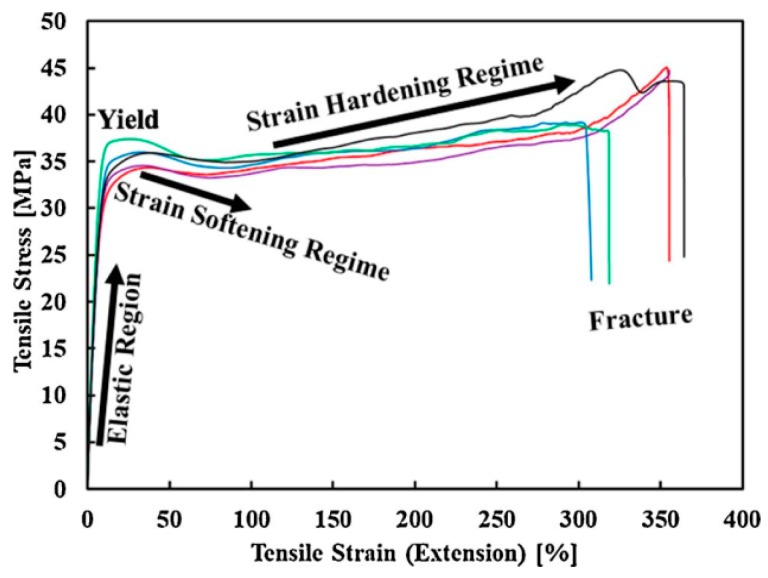
Large-strain hardening of five FFF Nylon6 samples with 100% infill density. Adapted from [136], with permission from © 2018 Elsevier.

Nylon is the first semi crystalline polymer in polyamides that is available at commercial scale for FFF. The preference of this material is justified by its good flexibility, least water absorption (<1.5%), good resistance to chemicals, good mechanical properties (Figure 8) and high fatigue resistance. 

Various commercialized printer makers have utilized Nylon to make their mark in the global market like Markforged [137]. Nylon12 by Stratasys Inc. has experimented with different layer thicknesses, types of nozzles, and build orientations. The on-edge orientation provides the highest strength of ~55 MPa followed by flat orientation with a close difference in tensile strength [68]. Post-treatment of Nylon doesn’t provide significant differences in tensile strength [54]. A recent research reports the addition of polyolefin elastomer grafted maleic anhydride (POE-g-MAH) to overcome the warpage [138]. During comparative analysis of stress-strain curves of FFF-based nylon, a wide range of strain hardening is found in literature as shown in Figure 8 [136]. Therefore, one of the future prospects of motivating the researchers regarding 3D printing of nylon is the enhancement and utilization of large strain hardening in potential applications.

In conclusion regarding single FFF polymers, PEEK is the only polymer that has been reported to have all three kinds of modifications, i.e., parametric, physical setup, and heated environment. Non-commercial PEEK holds the highest tensile strength followed by commercial PLA as shown in Figure 3 and Table 2. Parametric modifications are preferred for commercial PLA and Nylon to extract the superior properties as shown in Table 2. However, Nylon also reports both parametric and physical setup-based modifications to derive better results. PP also shows successful printing with both parametric and physical setup-based modifications. Variants of commercial ABS in a heated environment provide better properties as compared to non-commercial grades with optimal process parameters.

As a whole, the commercial polymers are explored more in terms of elastic modulus as compared to FFF single polymers made from injection or extrusion grade polymers (Figure 3). Therefore, it indicates a need to research the elastic properties of FFF parts printed by polymer filaments made of injection or extrusion grade. Furthermore, the effect of moisture, thermal and soil degradation on chemical structures of biodegradable materials (PLA, PCL) has not been properly investigated. This highlights the need for thorough chemical analysis of biodegradable FFF materials through Fourier transform infrared spectroscopy (FTIR), differential scanning calorimetry (DSC), thermosgravimetry analysis (TGA), etc.

### 2.2. Composites 

**Figure 9:**
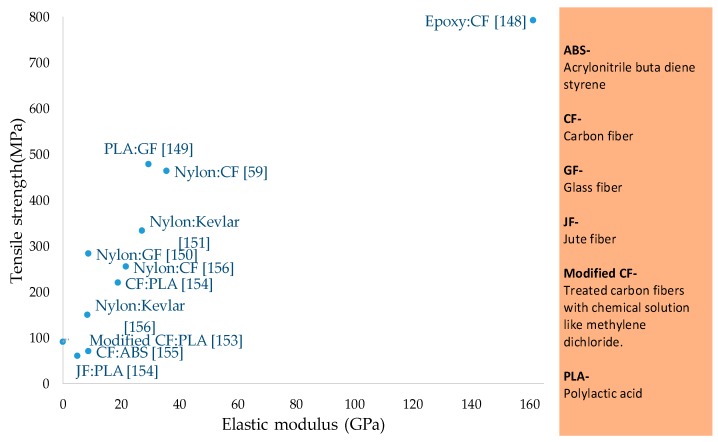
Tensile strength and elastic modulus of continuous fiber reinforced materials.

The contribution of reinforced composite materials is the most significant among all FFF materials as it enables printing of functional parts with highest mechanical strength (Figure 9 and Table 3, Table 4 and Table 5). The fiber reinforcement of different forms (continuous or discontinuous) and sizes improves the mechanical [139,140,141,142,143,144], thermal, and conductive properties [145,146,147]. Apart from strength, the reinforcements are added to overcome the non-printability regarding high co-efficient of linear thermal expansion [1,54]. The reinforced composites in FFF are made of either natural reinforcements (like fibers of palm, jute, hemp) or synthetic reinforcements (like carbon, glass, Kevlar, metal), as shown in Figure 2. The synthetic reinforcements are further classified into continuous and discontinuous reinforcements. Discontinuous synthetic reinforcements include fibers (short, micro, and nano), multiwalled nanotubes (MWNT), and powders. This section describes the significant research associated with continuous and discontinuous reinforced FFF polymers.

#### 2.2.1. Continuous Fiber Reinforced FFF Materials

**Figure 10:**
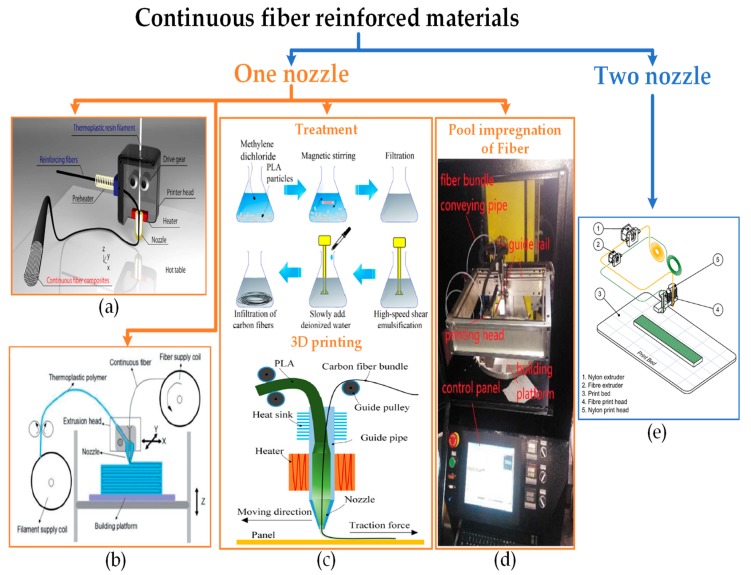
One nozzle and two nozzle illustration: (**a**) feeding heated fiber [154]; (**b**) feeding non-treated fiber, adapted from [61], with permission from © 2016 Elsevier; (**c**) feeding treated fiber with methylene dichloride and PLA pellets, adapted from [153], with permission from © 2016 Elsevier; (**d**) passing fiber from pool of melt, adapted from [148], with permission from © 2018 Elsevier; and (**e**) separate feeding of filament and fiber from two separate nozzles, adapted from [152], with permission from © 2018 Elsevier.

Continuous fiber reinforced polymers are directly fed into the FFF setup to achieve impregnation with polymer matrix. The accumulative strength of the composite is based on the strength and adhesion of both fiber and polymer matrix. To achieve the adhesion between fibers and polymer matrix, two kinds of physical setups are used in research. The pertinent setups are categorized with respect to the number of nozzles in this review, i.e., one nozzle for simultaneous impregnation (conventional FFF method) [148,149], and two nozzles for separate fiber and polymer matrix feeding [59,150,151,152] as shown in Figure 10. 

One-nozzle physical setups include four kinds of approaches for fiber impregnation as reported in the literature: (1) chemically treating the fibers before impregnation in synchronized fiber and polymer filament feeding [153], (2) heating the fiber to fuse fiber surface with polymer matrix in synchronized fiber and polymer filament feeding [154], (3) heating the polymer to create a melt pool to pass fiber through it in a non-synchronized fiber and polymer filament feeding [148,149], and (4) direct impregnation without any treatment or heating in synchronized fiber and polymer filament [61]. Each of these approaches achieves specific properties of tensile strength, flexural strength and ductility. Considering the highest tensile strengths (792.8 MPa [148] and 479 MPa [149]), the prominent approach is the impregnation of carbon fibers and glass fibers in epoxy melt and PLA melt pool inside the nozzle during non-synchronized feeding, respectively [148,149]. An additional three approaches, described below, further highlight different research gains in terms of understanding the nature of FFF composites. 

For example, heated synthetic carbon fibers achieve high diffusion of fibers into PLA matrix that is complemented by high tow strength of carbon fibers to provide >220 MPa tensile strength. The research also reports natural jute fibers with PLA matrix that results in high ductility instead of tensile strength, as compared to carbon fibers’ reinforced parts. Therefore, the brittle carbon fibers provide high strength and natural fibers exhibiting low brittleness attain higher ductility [154]. The non-treated carbon fibers with PLA matrix depicted the highest flexural strength of 335 MPa.

**Figure 11:**
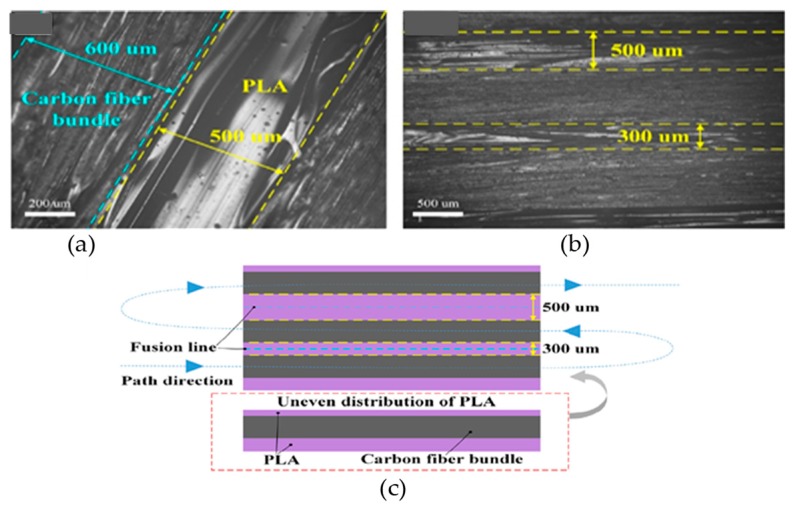
Surface morphology of 3D printed carbon fiber/PLA composite: (**a**) micrograph of carbon fiber bundle and PLA resin; (**b**) micrograph of PLA width between carbon fibers; and (**c**) schematic of the uneven distribution of PLA. Adapted from [153], with permission from © 2016 Elsevier.

However, the tensile properties behave not been investigated for non-treated fibers through a single nozzle [61]. On the contrary, the treatment of continuous carbon fibers with methylene dichloride solution before printing through a single nozzle with PLA matrix provides tensile strength of 91 MPa and flexural strength of 156 MPa. Though the polymer matrix was reported to distribute unevenly around continuous fiber (Figure 11), the flattening of extrudate by nozzle through extrusion pressure and surface treatment are presented as the reasons to enhance interfacial strength shared between fiber and polymer matrix [153].

**Figure 12:**
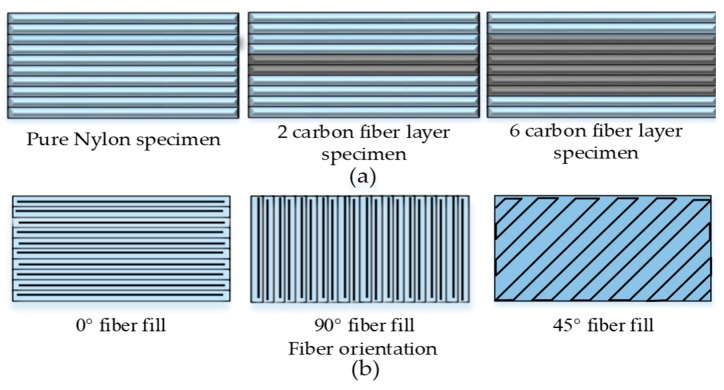
Illustration of two build strategies for FFF with two nozzles setups: (**a**) number and placement of carbon fibers in polymer matrix [59]; and (**b**) illustration of fiber fill orientation.

The main emphasis of the research reporting two nozzles is on the build strategy (Figure 12) that is comprised of a few important variables: (1) number of fiber layers, (2) placement of fiber layers in composite, and (3) raster angle of fiber lay-up and polymer matrix. The continuous uninterrupted stacking of a high number of fiber layers along with linear (isotropic) fiber and polymer raster orientation result in high mechanical strength [59,150,151,156].

#### 2.2.2. Discontinuous Fiber Reinforced FFF Materials

**Figure 13:**
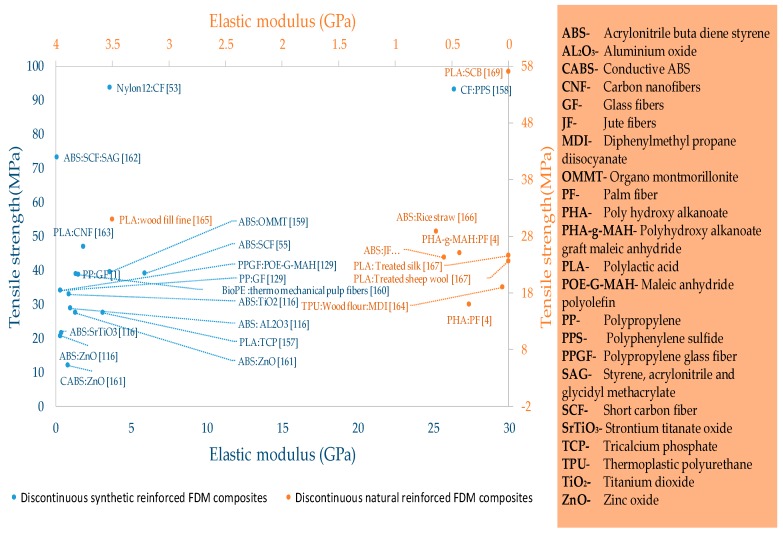
Tensile strength and elastic modulus of discontinuous fiber reinforced materials of fused filament fabrication.

**Figure 14:**
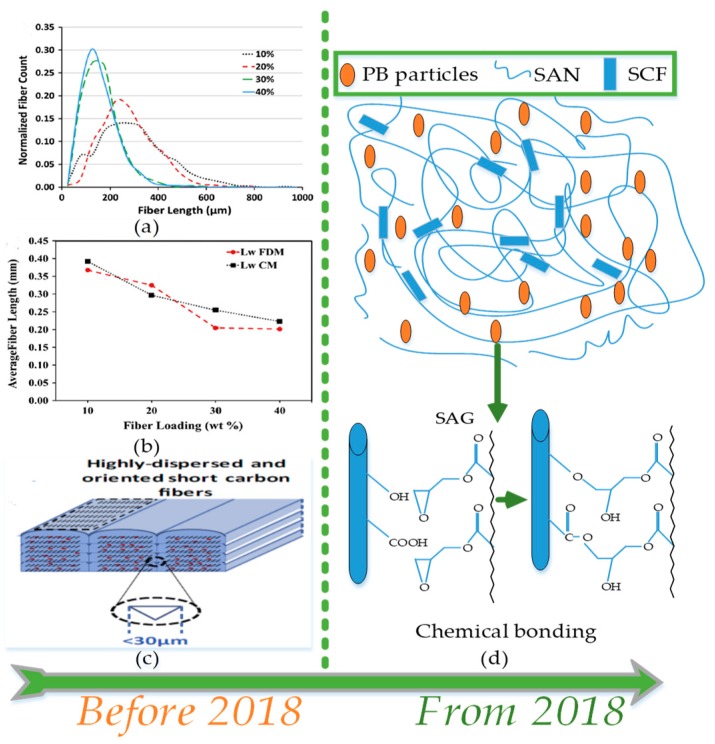
Development in research of discontinuous fiber reinforced FFF polymers before and from 2018: (**a**) fiber length distributions in FFF printed specimen; (**b**) weight average fiber lengths of FFF samples, and (**c**) dispersion of fibers in specific orientation. Adapted from [188], with permission from © 2014, Elsevier. (**d**) Illustration of chemical reaction of styrene acrylonitrile glycidyl methacrylate (SAG) on surface of short carbon fibers, adapted from [162], with permission from © Taylor & Francis.

The discontinuous reinforced composites are the most researched materials in reinforced FFF polymers as presented in Figure 13 and Table 4 and Table 5. It is noticed in this review that the type of discontinuous fibers (synthetic or natural) also brings significant effects on the properties of fiber-reinforced composites as shown in Figure 13. Furthermore, the functional characteristics of discontinuous reinforced composites are dependent on polymer matrix, adhesion between fibers and polymer matrix [53,55,129,161,162,163,175,176,177,178,179], fiber length distribution (FDL), orientation of fibers in matrix [53,158,180,181,182], packing densities [180], etc. Recent literature elaborates the difficulties associated with discontinuous fibers in the material processing stage that cause breakage of relatively long fibers during shear mixing in extrusion compounding and then 3D printing [183,184,185,186,187]. In this regard, the preliminary research focus is to compensate the uncontrollable breakage of long fibers through orienting the fibers in a dense FFF structure. The proper distribution (Figure 14a,b) and orientation (Figure 14c) attribute good tensile strength of about 70 MPa among discontinuous fibers reinforced composites [188]. 

The strategy of orienting the long fibers (12mm) with the help of high shear force during the FFF process results in the highest mechanical strength of 93.22 MPa for Big Area Additive Manufacturing (BAAM) that uses polypropylene sulphone (PPS) reinforced with carbon fibers [189]. The concentration of literature before 2018 is notable for the uniform dispersion to achieve homogenization of discontinuous entities (fibers, nano-tubes, powder) in the polymer matrix. On the contrary, recent developments have focused on the introduction of compatibilization of fiber surfaces with polymer as shown in Figure 14. Two recent publications regarding discontinuous fiber reinforced FFF composites report different methods of achieving compatibilization. One uses compatibilizer (styrene acrylonitrile glycidyl methacrylate, SAG) during ABS blending with short carbon fibers in a twin screw extruder (Figure 14d) [162], and the other treats ZnO powder with an ABS:acteone solution to form a surface layer of ABS for compatibilizing with ABS matrix [161]. 

Considerable efforts have been made to enhance the mechanical properties of natural fiber reinforced polymers (Table 5). However, in natural fiber reinforced polymers, the investigation of optimal printing process parameters is not the prime consideration as observed for single FFF materials. In fact, parameters such as nozzle temperature, layer thickness, infill percentage, bed temperature, and feed rate are mostly adopted from appropriate references (Table 5). Instead of process parameters, the preference is found for fiber concentration to study swelling, apparent draw ratio of filament during the filament making process, post-printing deflection, hydrophobic and hydrophilic properties, biocompatibility and biodegradability testing. Furthermore, this review enlists various methodologies that are used to develop natural fiber-reinforced composites (Figure 2). For example: (1) an old conventional method of using commercial (patent) reinforced filament [165], (2) blending non-processed reinforcements with neat polymers [166,190], (3) blending chemically processed reinforcements with neat polymers [167], (4) blending chemically processed fibers with laboratory prepared graft co-polymers [4], and (5) blending modified reinforcements with modifiers [164]. Each of these methodologies is discussed in further detail below.

Commercial wood-fill filament, made of recycled wood and binary polymer matrix of PLA and poly hydroxyalkanoate (PHA), is used to print the parts with a strength of 31 MPa. The high mechanical tensile strength is noted for samples printed at 0° orientation followed by compression with heating plates compared to simple 3D printed samples. The reason for enhancement in properties is the higher overlapping between beads at appropriate printing width that causes the reduction of porosities [165].

**Figure 15:**
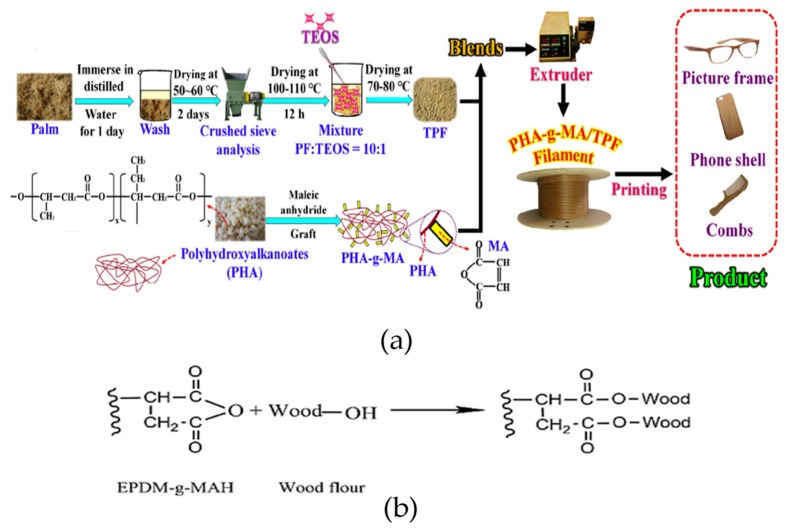
Two methodologies to develop natural fiber reinforced FFF materials: (**a**) in-laboratory prepared graft polymer for chemical processed natural palm fibers, adapted from [4], with permission from © 2017 Elsevier; and (**b**) modification of fibers matrix (TPU/WF) with modifier (EPDM-g-MAH) adapted from [164], with permission from © 2018 Elsevier.

A recent novel research provides a new direction of in-laboratory designed graft polymer matrix and chemically processed natural fibers that show high compatibility during blending. The maleic anhydride grafting on polyhydroxyalkanoate (PHA) and treatment of natural palm fibers with silane coupling agent and acetone provides (Figure 15a) better mechanical properties for graft matrix. In this research, solid-state carbon-13 nuclear magnetic resonance (CNMR) was used for the first time for FFF materials to justify the presence of grafting and good bonding between treated palm fibers (TPF) and maleic anhydride grafted PHA [4]. 

Another study presents the effects of different modifiers (EPDM-g-MAH, MDI, POE-g-MAH) on wood flour with thermoplastic polyurethane (TPU) as shown in Figure 15b. Though the reported tensile strength (~16 MPa) is not appreciable, this research strengthens the concept of using appropriate functionalized graft polymers that have an affinity towards particular natural fibers [164].

A unique study that developed their own kind of natural fiber reinforced FFF composites reports a hand-layup of chemically processed fibers (silk and sheep wool) in neat PLA matrix. The hand-layup of fibers is performed during the programmed stay time between neat printed layers. The maximum tensile strength of 23.66 MPa is recorded for a silk fiber-reinforced FFF composite [167].

A recent significant development for discontinuous natural reinforced FFF composite is the utilization of the characteristic weakness of natural fibers, i.e., hydrophilicity. High moisture absorption ability of natural fibers is a major technical obstacle that obscures the FFF parts to provide good resistance to moisture [191,192]. In contrast, the high moisture absorption capability makes the natural hygromorphic fibers to act like self-shaped wood [193,194,195], when incorporated as reinforcements in polymer matrix, making them suitable for 4D printing [196,197,198]. This development has broadened the applicable area of natural reinforced FFF composites.

Overall, reinforced composite materials possess the highest potential in terms of strength. However, it is noted that the research foci vary due to change in the type of the reinforced composites. For example, research on discontinuous reinforced composites concentrates on the uniform dispersion [188] and surface compatibility [162] of the reinforcements, and research on the continuous reinforced composites concentrates on the surface impregnation with resin [148,153,154]. Generally, process variables, physical setup modifications, and chemical processing play significant role in dispersion, compatibilization, or surface impregnation. The literature is scarce on printing in heated environments. Therefore, printing in the heated environments along with the enhanced chemical processing can be a potential area of discovery for future research. The rationale behind this is based on the significant improvements in single materials due to printing in the controlled ambient environment [83]. Furthermore, the stability of composites made with natural fibers and biodegradable materials is yet to be explored in terms of stability against moisture, thermal and soil degradation.

### 2.3. Blends

**Figure 16:**
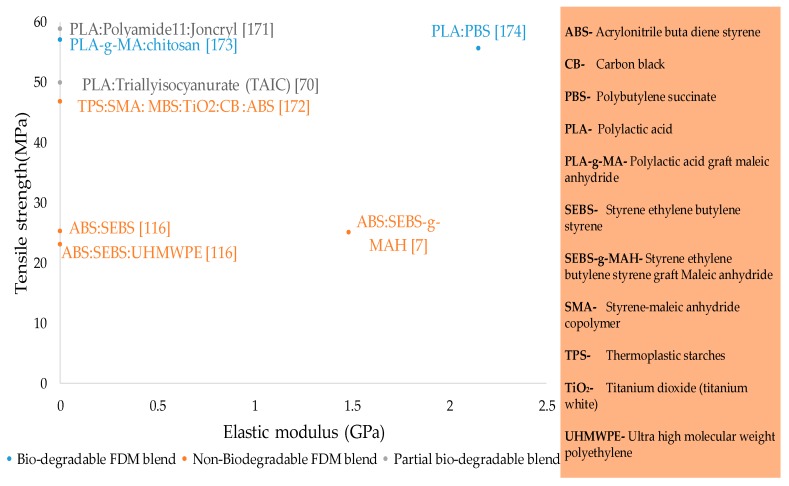
Tensile strength and elastic modulus of blend materials in fused filament fabrication.

Blending different polymers is an innovative concept, but very little research efforts have focused on this aspect of FFF (refer to Figure 16). Most FFF blends are listed in Table 6. The FFF blend materials are mostly made by either melt blending or reactive extrusion in a twin-screw extruder in the presence of additives like initiators, compatibilizers [7,171,173,199], molecular chain extenders [200], and plasticizers [116]. The majority of research on FFF-based blend materials reports the use of patent (commercial) graft [7] or non-graft [116] compatibilizers from different companies. One of the reasons for moving on to blends is the vulnerability retained by FFF structures due to their anisotropy. Substantial, but still insufficient, efforts are made to overcome the anisotropy through optimal combinations of parameters like layer thickness, air gaps, infill percentages, feed rate and printing speeds and raster angles [201,202], that cause large differences in tensile strength, particularly with Z-build orientation (vertical) [70,177,201,203,204,205,206,207,208,209].

The only reported strategy to overcome anisotropy is to develop new material systems by blending of printable materials with non-printable materials [116]. As mentioned earlier, a small amount of the research performed is associated with blends compared to single and composite materials. However, the significance of the limited research in blends overshadows their fewer numbers in terms of diversified functionalities such as enhancing inter/intra layer cross-linking, altering failure modes, improved biodegradable life, and an increase in ductility with reasonable strength. 

In this review, the FFF blends are categorized into three classifications as shown in Figure 2: (1) partial biodegradable blends, (2) non-biodegradable blends, and (3) biodegradable blends.

A recent publication regarding partial biodegradable blends is of PLA with radiation sensitizer (triallyl isocyanurate, TAIC) in the presence of organic solvent (dichloromethane). The polymer system, after being exposed to gamma rays, provides 49.9 MPa for 0° raster orientation. The results of PLA blend are preferable to control ABS (UTS = 31.1 MPa) due to the improved adhesion achieved through post-printing cross-linking between intercalated layers. The cross linking also helps to reduce the anisotropy in the FFF structure [70]. In a recent study [171], PLA has been blended with polyamide 11 in the presence of a novel compatibilizer known as Joncryl, which is a modified compatibilizer made of acrylic copolymer with epoxy functions. The results of this study showed the highest tensile strength (58.80 MPa) for 2% Joncryl among all FFF blends reported so far. 

Significant work regarding non-biodegradable blends reports two novel polymer blends of ABS with: (1) styrene ethylene butylene styrene (SEBS) and (2) ultra-high molecular weight polyethylene and styrene ethylene butylene styrene (SEBS:UHMWPE) [116,210]. Better mechanical strength (25.51 MPa) and ductility compared to pure ABS is obtained by binary polymer system (ABS:SEBS), even with poor interfacial adhesion that depicts the failure mode of a brittle nature in the corresponding research. However, the ternary polymer system (ABS:SEBS:UHMWPE) shows better layer bonding but with lower strength. The interfacial layer bonding is interpreted with complex viscosity measured by dynamic mechanical analysis (DMA) that reveals lower values for ternary blends than for control ABS. The low complex viscosity of ternary blends proves a high propensity to flow under shear that causes the extruded beads to spread on previous layers and hence reducing the anisotropy by filling the voids or air gaps [116]. The poor interfacial adhesion as reported in ABS:SEBS has been recently improved by the use of low molecular weight surface segregation additives (LMW-SuSAs) in a series of studies [211,212,213]. The most recent among those studies reports ABS with in-laboratory prepared SAN (styrene acrylonitrile), which results in better tensile strength (≈36 MPa) as compared to neat ABS [213]. 

**Figure 17:**
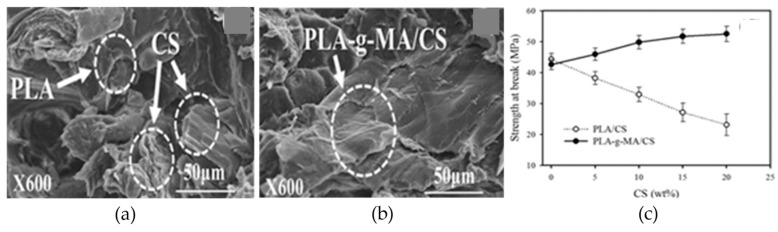
Scanning electron microscopy (SEM) images showing the distribution and wetting of CS in (**a**) PLA/CS (10 wt%); (**b**) PLA-g-MA/CS (10 wt%) composites; and (**c**) effect of CS content on the tensile strength at failure for PLA/CS and PLA-g-MA/CS composites. Adapted from [173], with permission from © 2016 Elsevier.

Biodegradable blends are also rare, similar to the other two types. One such type of blend is the polylactic acid grafted maleic anhydride (PLA-g-MA) with chitosan (CS), the second richest natural polymer. The better compatibilization of CS with PLA-g-MA compared to pure PLA (Figure 17a,b) strengthens the importance of polymer blend systems for the future. The research derived UTS of ~55 MPa for PLA-g-MA with 20% CS by weight as compared to 25 MPa of PLA-CS as shown in Figure 17c [173]. Another recent research reports the bimodal blend of high molecular weight PLA with an in-laboratory prepared low molecular weight PLA. The low molecular weight PLA acts as surface segregation additives that diffuse on the surface during the FFF process due to the long molecular chain [211].

As a summary of the literature regarding FFF materials, it is observed that the research on development of new materials depends upon the type of applications. A common research solution reported in the literature is the modification of existing polymers to achieve desired properties instead of making new monomers. The preliminary goal in modified FFF materials is the ability to print. This material development strategy leads existing research to have more composition by weight percent of existing printable materials (ABS, PLA, PP, Nylon) with low composition of non-printable materials (UHMWPE, SEBS-g-MAH, CS, triallylisocyanurate). Thus, the newly developed polymer blends have inherited functional groups from existing printable materials. Furthermore, existing materials like PEEK, ABS, PP, and Nylon are hard to print as they shrink and do not stick to the printing bed due to curl distortion. This requires some additional measures to make printability possible, including, heated environment, glued printing bed (surface), or incorporation of fibers. On the contrary as a key observation, PLA is the only material that does not provide any problem regarding printability in any form (single, composite, blend). 

**Figure 18:**
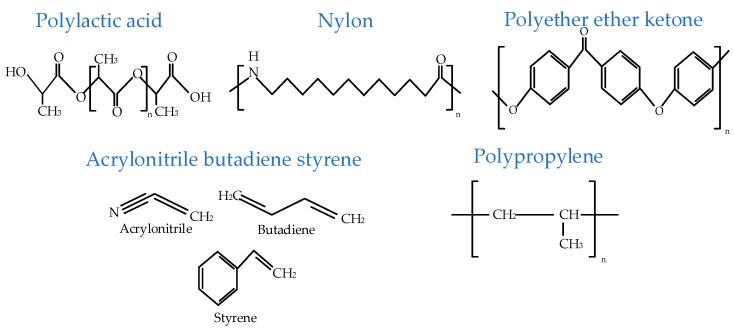
Chemical formulae of common FFF materials.

The simple analysis of chemical structural formulas shows two kinds of observable signs: (1) number of carbons in a monomer, and (2) number and type of functional groups. As shown in Figure 18, PLA has the simplest monomer with three carbons and one carboxylic functional group, Nylon has a long monomer with 11 carbons and two functional groups, PEEK has a long monomer with three aromatic rings (18 carbons) along with the carbonyl group, ABS is a ternary polymer with multi-functional groups including an aromatic group, polypropylene has the simplest monomer but with one of the longest chain configurations. The discussion leads to an understanding that polymers with a greater number of carbons, multi-functional groups, and complex groups (like aromatic) in a monomer are hard to print. However, polymers (like PLA) with a smaller number of carbon atoms in a monomer and one functional group are more suited to FFF printing. Therefore, there is a visible research gap for developing new, dedicated FFF polymers with short and single functional group-based monomers instead of blending printable with non-printable polymers. 

## 3. Summary and Conclusions 

Additive manufacturing is well-known for layer-by-layer manufacturing of complex parts at high precision. FFF/FDM is one of the oldest and the most widely used AM technology. Various aspects of FFF structures have been investigated in the literature, the majority of which have the prime consideration to achieve optimal or high strength (tensile, compressive, flexural) by either developing new materials or optimizing process factors or through a combination of both. This review presented the three process factors (process parameters, physical setup modifications, ambient temperature) and types of materials developed with time that are simultaneously employed to achieve improvement in tensile strength. 

**Figure 19:**
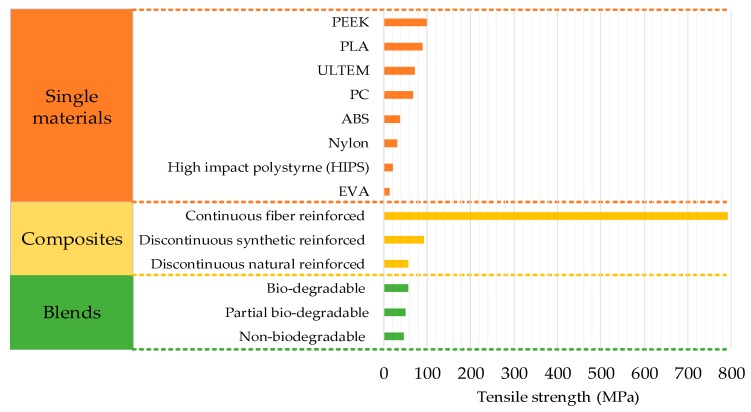
Strength range for different FFF materials.

A brief summary of the key findings in this review is as follows (Figure 19):

- The basic three types of materials, i.e., single, composites and blends, are analyzed in terms of modifications in process parameters, physical setups and environmental conditions. Furthermore, the above-mentioned three basic types are classified into the sub-categories to explain the modifications in materials that present their true capability.
- Single materials are categorized into commercial and non-commercial. Commercial materials are researched more than non-commercial materials. However, non-commercial materials have shown more potential to reach high tensile strength. For example, injection molding grade PEEK has the highest strength of 110 MPa as compared to 89.1 MPa for commercial PLA. Most research on single materials has shown a lack of physical modification of the printing system or temperature control. The research mostly comprises process variables’ optimization. The combination of physical setup modification and ambient temperature still has research potential for Nylon and ULTEM. Furthermore, biodegradable materials such as polycaprolactone (PCL), are not properly investigated for tensile properties as the literature generally reports the compression and flexural properties of PCL in FFF based medical applications.
- Composites are put into two main categories, continuous and discontinuous. Both of which are found in the form of natural and synthetic reinforcements. Discontinuous materials are researched more than continuous. The review provides further segregation of continuous synthetic reinforced composites, and synthetic and natural discontinuous composites with respect to their physical modification and chemical processing. Continuous materials are prominent with the highest strength achieved till now among all FFF/FDM materials. Furthermore, composites are mostly investigated with optimization with process variables and physical setup modifications. Therefore, the effect of ambient temperature is still not fully explored.
- Blends are segregated into three types: biodegradable, non-biodegradable, and partial biodegradable. However, blends are the least researched type of materials in FFF. The highest strength of 58.5MPa has been shown by the partial biodegradable blend. Like composites, blends are not researched in an ambient environment with temperature control. Therefore, this provides a novel area of research to combine this with the other aspects of blends.
- The review highlights the importance of developing novel polymers with less carbon atoms and functional groups instead of blending the printable contemporary materials with the non-printable materials. Furthermore, the review highlights numerous novel research areas regarding three types of materials (single, composites, and blends) as given in Table 7.

## Figures and Tables

**Table 1:** Fused filament fabrication (FFF) materials for different applications that are not generally investigated for tensile properties.

Domain	Applications	Materials
Medical	Scaffolds, Organs and Tissues	Poly caprolactone (PCL) [85], Poly(Ethylene Glycol) Terephthalate Poly(Butylene Terephthalate)(PEGT/PBT) [86], Chitosan/hydroxyapatite, Polyurethane [87], Poly l-lactide (L-PLA) [88], Corn starch/dextran/gelatin [89], Polylactic acid/Poly caprolactone [85], Chitosan/Hydroxyapatite (HA) [90], Chitosan/PLA/Keratine [91], Polyurethanes (PURs), Diisocyanate/Methylene diphenyl diisocyanate (MDI) [92], Polyols-polyether/PCL, Chain extender/Butanediol (BDO) [93].
Aerospace	Ceramic and metal filled parts	Zirconia/Wax [94], Polypropylene (PP)/Tricalcium phosphate (TCP), Polylactic acid/Hydroxyapatite (HA)/ceramic particles [95], Iron/nylon, Copper/Acrylonitrile butadiene styrene (ABS), Nylon 6/Al-Al_2_O_3_ [96,97], PC-ABS/Graphene
Electrical	Conducting products	ABS/Steel, PLA/Graphene/MWCNT [98], Polyurethane/MWCNT [99]
Unmanned air vehicle	Aerofoil, frame	Polyether imide (PEI) or ULTEM, Acrylonitrile styrene acrylate (ASA), Acrylonitrile butadiene styrene (ABS), Carbon fiber reinforced nylon [100].
Electronics	Sensors	Polylactic acid (PLA) [101], ABS, Wax blend, Nylon [83,102]

**Table 2:** Process factors for achieving high tensile strength of different single materials.

Material	Process Variables			Physical Setup	Environment	Tensile Strength (MPa)
	Variables	Set Values of Variables	Significant Variable			
PLA [62]	Build orientation	Flat, on-edge, upright Layer	Flat, 50 mm/s, and 0.06 mm	Not specific designed	Uncontrolled	89.1
	Layer thickness	0.06 mm, 0.12 mm, 0.18 mm, 0.24 mm.				
	Feed rate	20 mm/s, 50 mm/s, 80 mm/s				
PLA [113]	Strain rate	2.5 × 10^−4^ S^−1^, 1.25 × 10^−4^ S^−1^	2.5 × 10^−4^ S^−1^	Not specific designed	Uncontrolled	61.42
	Raster angle	0°, 45°, 90°	45°			
	Thermal comparison of material in different condition (for crystallinity)	As received filament, Extruded filament, Printed, Printed (annealed)	No significant difference in % crystallinity			
ABS [7]	Extrusion melt pump pressure	75 for MG47, 54 for MG94	Both grades	Not specific designed	Uncontrolled	34
	Two molecular weight grades	MG47 for high MW, MG94 for Low MW	MG47			
PEEK [117]	Infill percentage	20, 50, 100	Flat and 100% infill	Not specific designed	Uncontrolled	≈100
	Build orientation	Flat, vertical				
PEEK [121]	Two molecular weight grades	OPTIMA LT3 (low MW),VICTREX 450G (high MW)	VICTREX 450G 14%	Two kinds of setup Syringe based Filament based	Heated plate Lamp heated atmosphere	75.06
	Average Porosity %	14%, 31%				
	Printing speed	0 to 120 mm/min				
	Extrusion speed	0 to 120 mm/min				
	Nozzle diameter	621.052 µm, 512.03 µm, 407.96 µm				
PEEK [124]	Printing methods	Line printing, Plane printing	Plane printing	Pellet printer, Glass and steel plate		98
PEEK [52]	Build orientation	Flat, vertical,	Flat and 0°			82.5
	Raster angle	0°, 90°				
PP [1]	Infill percentage	20%, 60% and 100%	100% 0° 0.2	Custom extrusion head. Scrubbed glass bed with alcohol treatment [126]	Uncontrolled	36
	Orientation	45°, 0°, 90°, crossed 45° (±45°) and crossed 0°–90°				
	Layer thickness	0.20 and 0.35				
Nylon [68]	Build orientation	Flat, on-edge, supright (vertical)	T16 On-edge	Nozzle size T12, T16, T20	Uncontrolled	~55

**Table 3:** Process factors for achieving high tensile strength for continuous fiber reinforced composites.

Materials	Process Variables			Physical Setup	Environment	Tensile Strength (MPa)
	Variables	Set Values of Variables	Significant Variable			
CF:ABS [155]	Different printers	Makerbot replicator, CubeX, Afinia and Solidoodle 3	All printers have significance Flat samples have maximum UTS	No	No	70.69
	Build orientation	Flat, Vertical				
Modified CF:PLA [153]	Pre-printing treatment of CF	Methylene dichloride solution with 8% PLA particles for CF.	Treatment	Customized	No	91
JF: PLA CF: PLA [154]	Pre-printing heating of continuous CF	210 °C	Carbon fiber	No separate mechanism for pulling CF. Nichrome wire heater attached with printing head for heating CF.	No	220 (CF) 60 (JF)
	Fiber types	Carbon fiber (CF) and Jute fiber (JF)				
Epoxy: CF [148]	Epoxy pool impregnation of CF	Epoxy pool impregnation	Epoxy pool impregnation	Customized setup	No	792.8
	Printing schemes	Lamina, Honey comb, Grid (not for UTS)				
Nylon: CF Nylon: Kevlar [156]	Fiber types	Carbon fiber, Kevlar fiber	Nylon: CF 0°	No (Mark One 3D printer)	No	254.8 (CF), 150.2 (Kevlar)
	Raster orientation	Orientations for Nylon: Kevlar (0°, ±45°), Nylon:CF (0°)				
Nylon:CF [59]	Fiber build strategy with discontinuity in fiber layup each path	Sandwiched Carbon fibers in middle of 10-layer specimen, i.e., 2 layers, and 6 layers	6 CF layer	No (MarkForged company printer)	No	464.4
PLA:GF [149]	Pool of PLA	Pool of PLA	Pool of PLA 49.3 0.3	Customized	No	479
	Fiber composition %	49.3,46.3,40.18,35.14,28.78,22.74				
	Extrusion width (mm)	0.22,0.25, 0.35, 0.4,0.5,0.6,0.8				
Nylon: GF Nylon: Kevlar [150]	Fiber composition %	25% and 50%	50% Isotropic 0°	Non-commercial two nozzle printing head	No	283.5
	Fill type	Isotropic and Concentric				
	Fill type category 1) Isotropic fill type 2) Concentric fill type	0°, 45°, and 90° 4 layers 8 layers, and 12 layers				

**Table 4:** Process factors for achieving high tensile strength for discontinuous synthetic reinforced materials.

Materials	Process Variables			Physical Setup	Environment	Tensile Strength (MPa)
	Variables	Set Values of Variables	Significant Variable			
PLA: TCP [157]	Specimen size	1:1, 1:2	1:2 225 C	No		27.5
	Printing temperature	215 °C, 225 °C, 235 °C				
PP:GF [1]	Infill degree %	20%, 60%, 100%	Infill 100% 0° 0.35 mm	Customized printer	No	39
	Raster orientation	45°, 0°, 90°, crossed 45° (±45°), 0°–90°				
	Layer thickness	0.2 mm, 0.35 m				
ABS: TiO_2_, ABS: ZnO, ABS: SrTiO_3_, ABS:AL2O_3_ [116]	Build orientation	Flat, Vertical	Flat TiO_2_	No	No	32.9 (ABS:TiO_2_) 20.7 (ZnO) 21.6 (ABS:SrTiO_3_) 28.8 (ABS:AL2O_3_)
	Type of fillers	TiO_2_, No, SrTiO_3_, AL2O_3_				
CF:PPS [158]	Raster orientation	0° (longitudinal), 90° (transverse)	0°	No	No	93.22
ABS: OMMT [159]	Laboratory based OMMT (treated) content %	1%, 3%, 5%	5%	No	No	39.48
BioPE: TMP [160]	Laboratory prepared thermos- mechanical pulp fibers (TMP) %	0%, 10%, 20%, 30%	30%	No	No	38.72
ABS: ZnO CABS: ZnO [161]	Type of polymer matrix	ABS, CABS	ABS 100 % Line	Powder ZnO deposition by dispenser during printing	No	27.5 (ABS:ZnO) 12 (CABS:ZnO)
	Infill density	50%, 75%, 100%				
	Infill pattern	Line & rectilinear with 45° raster				
PPGF: POE-g-MA [129]	Layer thickness	0.1mm and 0.4 mm	0.1 mm 20%	Laboratory made PP tape for heating bed	No	34
	POE-G-MA contents %	10%, 20%, 30%				
ABS: SCF: SAG [162]	SAG content %	0%, 1%, 3%, 5%, 7%	5%	No	No	73.3
ABS: SCF [55]	Type of reinforcement	Short carbon fibers (SCF), Carbon nanotubes (CNT)	SCF 0°	No	No	39.05
	Raster angle	45°/-45°, 0° and 90°				
PLA: CNF [163]	Nozzle geometry	Circle, and square	Square (less voids) 0.5%	No	No	47
	CNF contents %	0.5%, 0.1%				
Nylon12:CF [53]	CF contents%	0%, 2%, 4%, 6%, 8%, 10%	10% 0°	No	No	93.8
	Raster angle	0°, 90°				

**Table 5:** Process factors for achieving high tensile strength for discontinuous natural fiber reinforced materials.

Materials	Process Variables			Physical Setup	Environment	Tensile Strength
	Variables	Set Values of Variables	Significant Variable for Highest Strength			
ABS:JF [116]	Build orientation	Flat, Vertical	Flat TiO_2_	No	No	24.25 (ABS: JF)
	Type of fillers	Jute, TiO2, ZnO, SrTiO_3_, AL2O_3_				
TPU: Wood flour: MDI [164]	Wood flour contents %	10%, 20%, 30%, 40%	MDI	No	No	19
	Types of modifiers	EPDM-g-MAH, POE-g-MAH, chitosan (cs), polyethylene glycol (PEG), diphenyl methyl propane di-isocyanate (MDI)				
PHA-g-MAH:PF [4]	Treated palm fiber with Silane coupling agent.	Treated palm fibers (PF)	20% PHA-g-MAH	No	No	25
	PF composition	10%, 20%, 30%, 40%				
	Type of polymer matrix	Laboratory prepared PHA-g-MAH, PHA				
PLA: wood fill fine [165]	Sample width %	100%, 200%, 300%	100% 0°	No	No	31
	Raster angle	0° and 90° (rectilinear infill)				
ABS: Rice straw [166]	Number of contours	1, 2	2 15%	No	No	28.89
	Rice straw content %	5%, 10%, 15%				
PLA: Silk [167]	Types of fibers	Sheep and Silk wool (chemically treated)	Silk 4 100% 0°/90°	No specific physical change. Just provided stay time between layers	No	24.58 (PLA: Silk) 23.63 (PLA: Sheep wool)
	Number of laminates	2, 3, 4				
	Infill density	20%, 60%, 100%				
	Raster angle	0°/90°, 45°/135°, 30°/120°				
PP: Harakeke PP:Hemp [168]	Types of fibers	Harakeke, hemp	20% Harakeke	LDPE & PP bed. warpage in glass	No	24 (PP: Harakeke) 16 (PP: Hemp)
	Fiber composition	10%, 20%, 30%				
PLA: Sugarcane bagasse [169]	Raster angle	0°/0°, 45°/-45°, 0°/90°, 90°/90°	45°/45° (only provided tensile strength at 45°/−45°) Sugar cane bagasse fibers	No	No	57
	Sugar cane bagasse fiber composition	3%, 6%, 9%, 12%, 15%				
	Raw sugarcane bagasse composition	3%, 6%, 9%, 12%, 15%				

**Table 6:** Process factors for achieving high tensile strength for blend materials.

Materials	Process Variables			Physical Setup	Environment	Tensile Strength (MPa)
	Variables	Set Values of Variables	Significant Variable			
PP: SEBS [170]	Composition of PP:SEBS	20:80, 40:60, 60:40	7.5 phr carbon black	PP print bed	No	18 (7.5 phr) 14 (40PP:60SEBS)
	Carbon black in 40PP:60SEBS	0-15 parts per hundred rubber (Phr)				
	Injection molding	40PP:60SEBS				
PLA:PA11:Joncryl [171]	Composition of Joncryl (modified acrylic copolymer with epoxy functions)	0%, 1%, 2%, 3%	80:20:2 (PLA:PA11:Joncryl) Injection moulding	No	No	58.8
	Different processes	Injection moulding, FDM				
ABS:SEBS [116]	Build orientation	Flat, Vertical	Flat TiO_2_ 95:5 90:10:10	No	No	25.5 (ABS: SEBS) 23.07 (ABS: UHMWPE: SEBS)
	Type of fillers	SEBS, UHMWPE: SEBS, Jute, TiO_2_, ZnO, SrTiO_3_, AL2O_3_				
	Composition	ABS: SEBS (95:5, 80:20) ABS: UHMWPE: SEBS (90:10:10, 75:25:10)				
TPS:ABS:SMA: MBS:TiO2:CB [172]	Types of polymers Polymers	Styrene maleic anhydride (SMA), methyl- methacrylate butadiene styrene (MBS), TiO_2_, pigment CB	SMA 30ABS:70%:1SMA:0% TiO_2_:0%CB	No	No	46.8
	Composition	SMA (1%), MBS (1%, 2%), TiO_2_ (0%, 5%), CB (0%, 5%),				
ABS:SEBS-g-MAH [7]	Grades of ABS	MG47, MG940 (w.r.t molecular weight)	MG94 60 mm/s 75%:25%	No	No	25.09
	Feed rate	30 mm/s and 60 mm/s				
	Composition of ABS: SEBS-g-MAH	75:25, 50:50, 25:75 One additional for MG94 in 10:90				
PLA-g-MA: Chitosan [173]	Types of polymers	PLA, Laboratory prepared PLA-g-MA,	PLA-g-MA 20%	No	No	57
	Chitosan (CS) composition%	5%, 10%, 15%, 20%				
PLA-PBS [174]	PBS content %	20%, 40%, 60%, 80%	20%	No	No	55.6

**Table 7:** Novel areas of research for different types of FFF materials.

Material	Novel Area/s to Explore
PLA	Effects of moisture, thermal and soil degradation on chemical structure and tensile strength
ABS	-
Nylon	Large-strain behavior to be explored in structural applications
PP	Effects of printing in heated environment
PC	Effects of post-printing thermal treatment
PEEK	-
Composites	1. Printing in heated environment 2. Stability of biodegradable composites against moisture and soil degradation 3. Optimal composite properties considering process (printing) temperature as a variable.
Blends	1. Printing in heated environment 2. Stability of blends against post printing thermal degradation 3. Optimal blend properties considering process (printing) temperature as a variable.
